# Effect of Long-Term Contraception with Altrenogest in Dolphins (*Tursiops truncatus*)

**DOI:** 10.3390/ani16030399

**Published:** 2026-01-27

**Authors:** Vincenzo Cicirelli, Alice Carbonari, Lucrezia Forte, Roberta Carreca, Rocio Canales, Teresa Fernandes, Letizia Fiorucci, Annalisa Rizzo

**Affiliations:** 1 Department of Veterinary Medicine, University of Bari Aldo Moro, S.P. 62 per Casamassima km. 3, 70010 Valenzano, BA, Italy; vincenzo.cicirelli@uniba.it (V.C.); lucrezia.forte@uniba.it (L.F.); r.carreca@studenti.uniba.it (R.C.); annalisa.rizzo@uniba.it (A.R.); 2 Mundomar, 03503 Benidorm, Spain; veterinario@mundomar.es; 3 Jardim Zoológico de Lisboa, 1549-004 Lisbon, Portugal; teresalf@zoo.pt; 4 Aspro Parks Canarias SL, 38660 Santa Cruz de Tenerife, Spain; lfiorucci@aspro-ocio.es

**Keywords:** *Tursiops truncatus*, Altrenogest, hormonal profile, reproductive diseases, contraception

## Abstract

Altrenogest is a synthetic progestin used as a contraceptive method in various animal species, including bottlenose dolphins. This is a retrospective study based on the analysis of data collected between 2020 and 2025. Eighteen female dolphins (*Tursiops truncatus*) from four zoological parks on the Iberian Peninsula were included, treated with the progestin for variable periods. The animals were monitored through ultrasound examinations, behavioral observations, and hormonal assays for progesterone and estrogen. The results showed statistically significant changes in hormone levels, with a significant decrease in serum progesterone and a significant increase in estrogen levels in treated dolphins compared to untreated ones. These findings suggest that Altrenogest inhibits LH secretion, while it does not appear to directly affect FSH, allowing some degree of follicular activity to persist. Despite its demonstrated contraceptive efficacy, prolonged use of the drug was associated with the development of pyometra in four individuals and follicular cysts in three individuals. These results highlight the effectiveness of Altrenogest in reproductive management of dolphins, while also emphasizing the need for careful evaluation of treatment duration and the potential risk of long-term adverse effects.

## Simple Summary

In zoological facilities, controlling reproduction in bottlenose dolphins is essential to ensure animal welfare and appropriate population management. Altrenogest is a synthetic hormone widely used to prevent pregnancy in female dolphins, but information on its long-term effects is still limited. In this retrospective study, we evaluated the impact of prolonged Altrenogest administration in 18 female bottlenose dolphins housed in four zoological parks. Hormonal monitoring, ultrasound examinations of the reproductive system, and behavioral observations were analyzed over several years. The treatment effectively suppressed reproductive behavior and ovulation, as indicated by consistently low progesterone levels. However, dolphins receiving long-term Altrenogest treatment showed a higher occurrence of reproductive disorders, including uterine infections (pyometra) and ovarian follicular cysts, compared with untreated animals. These findings suggest that although Altrenogest is an effective contraceptive method, its prolonged use may increase the risk of reproductive health problems. Careful evaluation of treatment duration and regular clinical monitoring are, therefore, crucial to ensure the long-term health and welfare of dolphins under human care.

## 1. Introduction

The bottlenose dolphin (*Tursiops truncatus*) is the most common cetacean maintained under human care and is generally described as seasonally polyestrous [1,2]. However, other authors report a polyestrous pattern interspersed with anestrous periods lasting one or even two years [3]. Reproductive seasonality may also vary depending on the geographical origin of the females or of the founder individual from which a captive population was established [4]. Most estrous cycles occur from spring through autumn, although births have been documented in every month of the year. A very important issue is that assessing estrogen fluctuations is challenging without serial blood sampling, which complicates the characterization of estrous and ovulatory patterns. Ultrasonography remains the preferred method for monitoring the estrous cycle and predicting ovulation [2]. In addition, daily collection of urinary or fecal samples, represents the most reliable non-invasive approach for identifying endocrine–ovarian relationships. In *Tursiops truncatus*, the estrous cycle is estimated to last between 21 and 42 days [5], with estrogen levels elevated for approximately 5–7 days [6]. Another study defined cycle duration as the interval between two successive LH peaks or between peaks of urinary estrogen conjugates (ECs) and urinary progesterone (UP) [5]. Periods of anestrus unrelated to pregnancy or lactation have been reported, with ovulatory pauses lasting up to 27 months, although the underlying causes remain unclear [7]. Fertility control is a common concern in facilities housing marine mammals, as bottlenose dolphins can be highly fertile under managed care. For more than two decades, Altrenogest (Regu-Mate^®^, Merck Animal Health, Rahway, NJ, USA), a synthetic progestin, has been used in cetaceans both for synchronization and as a contraceptive [5]. Altrenogest acts by binding to hypothalamic receptors and inhibiting the pituitary release of LH and FSH. Originally developed for use in mares [8], it was subsequently shown to be effective in sows as well [9]. The recommended contraceptive dosage across species is 0.044 mg/kg once daily, administered orally [10]. Long-term use of Altrenogest has been reported without apparent adverse effects on fertility in killer whales [1], Pacific white-sided dolphins (*Lagenorhynchus obliquidens*), and bottlenose dolphins [11]. This paper is a retrospective study on the use of Altrenogest in female *Tursiops truncatus* dolphins kept under human care. The study covered the period between 2020 and 2025, examining data collected at four zoos located in the Iberian Peninsula and Canary Islands. The objective was to evaluate the safety of using Altrenogest, through data obtained from hormonal, ultrasound, and behavioral monitoring, to help optimize reproductive management strategies in cetaceans in a controlled environment.

## 2. Materials and Methods

### 2.1. Ethical Statement

All procedures were carried out in accordance with institutional guidelines for animal welfare, with the Animal Welfare and Experimentation Committee of the University of Bari under protocol number 28/24.

### 2.2. Animals and Experimental Design

**Figure 1:**
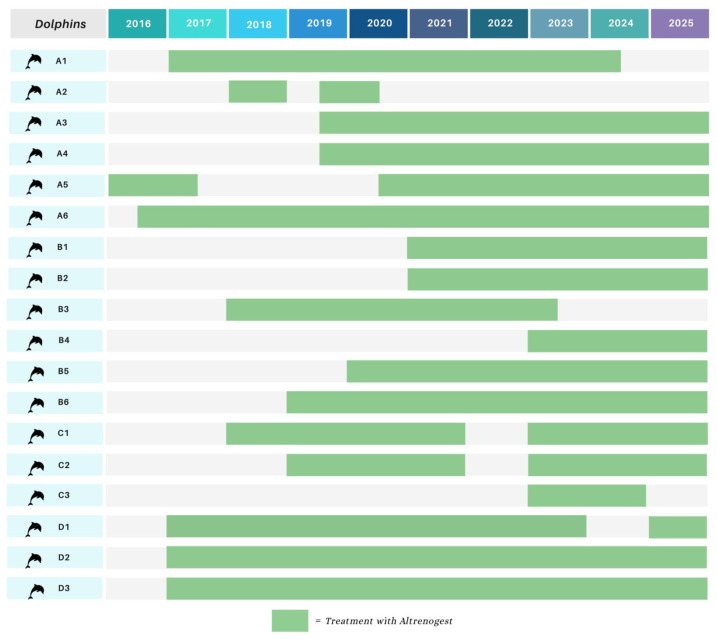
Treatment periods from 2016 to present of enrolled dolphins.

This is a retrospective study based on the analysis of data collected between 2020 and 2025. The study was conducted on a total of 18 female *Tursiops truncatus*, hosted in four zoos located on the Iberian Peninsula and Canary Islands. Animal husbandry is similar in the four zoos, both in terms of feeding and management. Specifically, diet is adjusted according to individual needs, considering factors such as age, physiological or pathological status, sex, and seasonal variations. The diet is structured based on a controlled intake of kilocalories and kilograms and includes various fish species, including *Clupea harengus* (herring), *Mallotus villosus* (capelin), *Sprattus sprattus* (sprat), *Micromesistius poutassou* (blue whiting), *Scomber scombrus* (mackerel), *Atherina boyeri* (smelt), and *Loligo* spp. (squid). All animals also undergo regular veterinary checks as part of preventive medicine programs [12]. A key component of the preventive health program is the training of medical behaviors, allowing dolphins to voluntarily participate in medical assessments such as the collection of biological samples and the use of non-invasive methods, including ultrasonography. Reproductively, fertility is controlled using Altrenogest (Regu-Mate^®^, Merck Animal Health, Rahway, NJ, USA), administered orally at a dose of 0.044 mg/kg once daily. Figure 1 shows the treatment periods for all study animals from 2016 to present.

The females included in the study ranged in age from 8 to 57 years at the time of data collection. During the years considered (2020–2025), the following were analyzed:

- Serum progesterone and estradiol levels, obtained from routine hormone monitoring (at least twice per year).
- Ultrasound findings of the reproductive tract of the animals, concomitant with hormone monitoring.
- Any reproductive pathologies detected: Pyometra and follicular cyst. Pyometra has been defined as an infection of the uterus with echographically detectable fluid accumulation, thickening of the walls, and alterations in the blood inflammatory panel. Follicular cysts are defined as follicular structure ≥10 mm in diameter that persists for at least 10 days without evidence of ovulation [13].
- Behavioral observations made by the trainers.

All collected data were assigned to two groups based on the presence/absence of hormonal conditioning at the time of observation:

- Altrenogest: for conditioned dolphins.
- Control: for unconditioned dolphins (considering a washout period of 60 days to encompass at least one complete estrous cycle and to allow the resolution of residual endocrine effects of the progestin on the hypothalamic–pituitary–gonadal axis).

### 2.3. Blood Sampling and Hormonal Analyses

A sample was taken every six months from each animal. Blood sampling was performed from the superficial veins of the caudal fins using a 21G butterfly needle connected to a 10 mL syringe. The blood was collected in a tube with separator gel and then centrifuged (3500× *g* for 10 min) to obtain serum. All serum aliquots were stored at −20 °C until analysis. All blood samples were collected from animals fasting for at least 8 h. The mini VIDAS apparatus (Biomérieux, Marcy-l’Étoile, France) was used to measure progesterone (P_4_) and estradiol (E_2_). Specifically, the immunoenzymatic competitive method was used with final detection via ELFA (Enzyme-Linked Fluorescent Assay), with a detection limit of 0.25 ng/mL (0.80 nmol/L) for P_4_ and 9.0 pg/mL (33.0 pmol/L) for E_2_.

### 2.4. Ultrasound

Ultrasound examinations were performed monthly using a General Electric ultrasound system (Versana Active, GE HealthCare, Chicago, IL, USA) with a 2–5 MHz convex probe, in accordance with the methodology described by Brook (2001) [14]. Ultrasound images of the ovaries (measurements, presence/absence of functional structures) and the uterus (measurements, contents) were collected, and findings consistent with uterine and ovarian pathological conditions were also recorded.

### 2.5. Behavior

The animals involved in the study are cared for daily by marine mammal specialists, who ensure their physical and psychological well-being through cognitive enrichment, appropriate nutrition, and structured social interactions. In this study, these animal care specialists also played a key role in collecting behavioral data. Specifically, incidents of sexual behavior and any mating observed during training sessions or during daily monitoring of the animals were recorded and reported.

### 2.6. Statistical Analysis

The assumptions of normal data distribution and homogeneity of variances were assessed using the Shapiro–Wilk test. Data were analyzed by analysis of variance (ANOVA) using the General Linear Model (GLM) procedure in SAS software (version 9.3; SAS Institute Inc., Cary, NC, USA). Initially, the effect of the experimental group alone was evaluated according to the following model:

y_ij_ = μ + α_i_ + G_j_ + ε_ij_,

where y_ij_ represents all parameters as dependent variables, μ is the mean, α_i_ is the single animal random effect (1, …, 36), G represents the effect of the jth experimental group (j = 1, 2), and ε_ij_ is the error. Pairwise comparisons between diets within the same time point were performed using the Bonferroni test.

Subsequently, the effects of the experimental group, sampling time, and their binary interaction were evaluated according to a second model:

y_ijk_ = μ + α_i_ + G_j_ + T_k_ + (G × T) _jk_ + ε_ijkl_,

where y_ijk_ represents all parameters as dependent variables, μ is the mean, α_i_ is the single animal random effect (1, …, 36), G represents the effect of the jth experimental group (j = 1, 2), T represents the effect of the kth sampling time (k = 1, …, 11), G × T represents the effect of the binary interaction between the two independent variables (jk = 1, …, 22), and ε_ijkl_ is the error. Pairwise comparisons between groups within the same time point were performed using the Bonferroni test. Tukey’s post hoc test for repeated measures was then applied to determine differences across time. Serum hormone concentrations (estradiol and progesterone) are expressed as least square means ± standard error of the mean (SEM). Statistical significance was declared at *p* < 0.05.

## 3. Results

Between 2020 and 2025, a total of 192 blood samples were collected from the Altrenogest group (average of 2 samples/animal/year) and 198 samples from the Control group (average of 2 samples/animal/year). Sampling was homogeneous between groups in terms of frequency and seasonality. In the last three sampling periods, 4 years, 4 years and 6 months, and 5 years, one, one, and four dolphins, respectively, were not considered in the Altrenogest group due to the onset of pyometra.

All animals treated with Altrenogest showed no sexual behavior and did not mate, demonstrating the effectiveness of hormonal conditioning. Analysis of serum estradiol and progesterone levels showed significant differences between the Control group and the Altrenogest group, as reported in Table 1.

Treatment with Altrenogest resulted in a marked increase in estradiol concentrations (14.11 vs. 9.03; *p* < 0.0001) and a significant reduction in progesterone levels (0.31 vs. 3.01; *p* < 0.0001) compared to the Control group. The effect of the group, sampling time, and their binary interaction on serum estradiol and progesterone levels were evaluated, and the results are shown in Table 2.

Estradiol levels remained consistently lower in the Control group than in the Altrenogest group at all points, and no significant differences were detected for either the time effect or the group × time interaction (*p* = 0.9935 and *p* = 0.9993). Progesterone levels were higher in the Control group, while in the Altrenogest group they remained low throughout the observation period. Again, the effects of time and group–time interaction were not statistically significant (*p* = 0.0782 and *p* = 0.0731).

**Figure 2:**
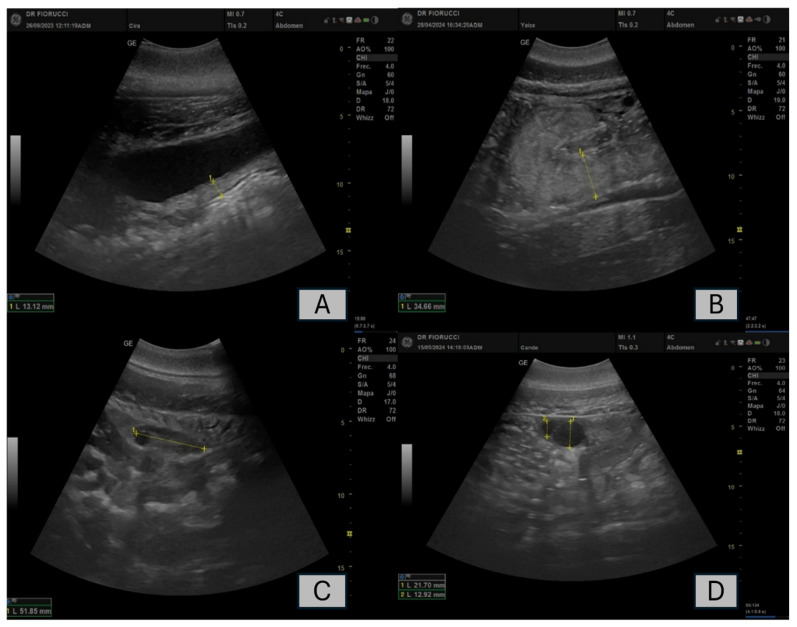
Ultrasonographic evaluation of dolphin reproductive tract. Panel (**A**) shows the uterine body of a dolphin with pyometra, with thickening of the uterine wall (13.2 mm) and the presence of fluid. Panel (**B**) shows the uterine horn of a dolphin with hyperechoic particulate material and a total horn thickness of 34.66 mm. Ovarian measurements in two different scanning planes (panels (**C**,**D**)). Panel (**C**) shows the ovary with a diameter of 51.85 mm, while panel (**D**) displays cystic structures with completely anechoic content, measuring 21.7 mm and 12.9 mm.

The reproductive disorders observed were pyometra and follicular cysts (Figure 2). Analysis of the incidence of these disorders revealed clear differences between the two groups (Altrenogest and Control).

**Figure 3:**
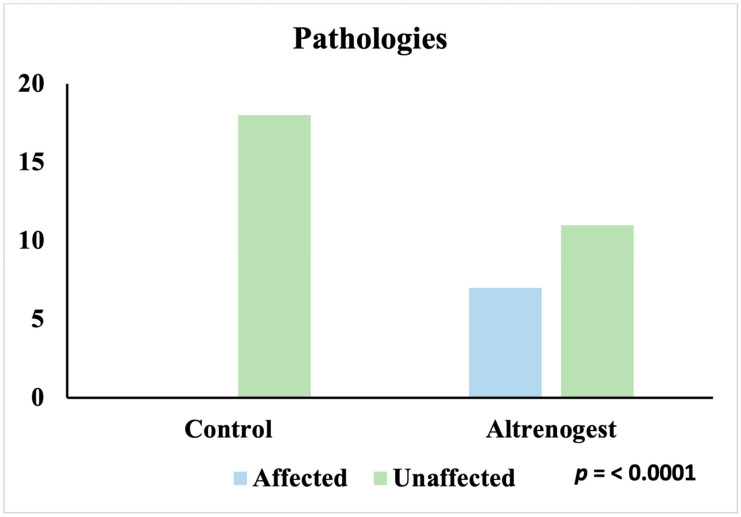
Incidence of pyometra and follicular cysts in Control and Altrenogest groups.

**Figure 4:**
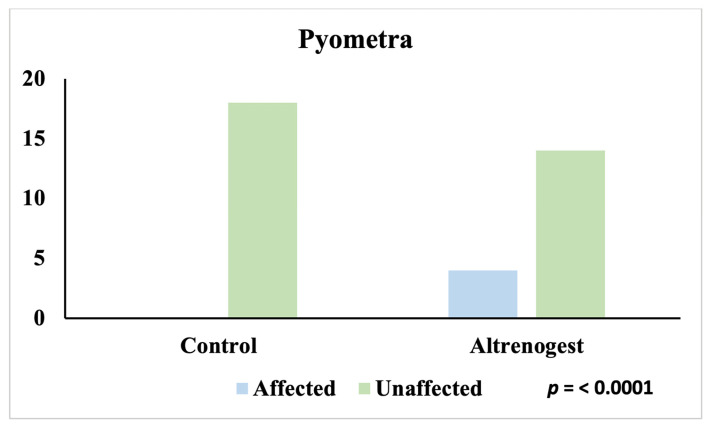
Incidence of pyometra in Control and Altrenogest groups.

The overall assessment of the two conditions confirms that only animals treated with Altrenogest were affected (7/18; 39%), while the entire Control group was unaffected (18/18; 100%) (*p* < 0.0001) (Figure 3). Analyzing the individual pathologies separately, the same trend is confirmed: pyometra was found only in the Altrenogest group (4/18; 22%), while all subjects in the Control group were unaffected (18/18; 100%) (*p* < 0.0001) (Figure 4).

**Figure 5:**
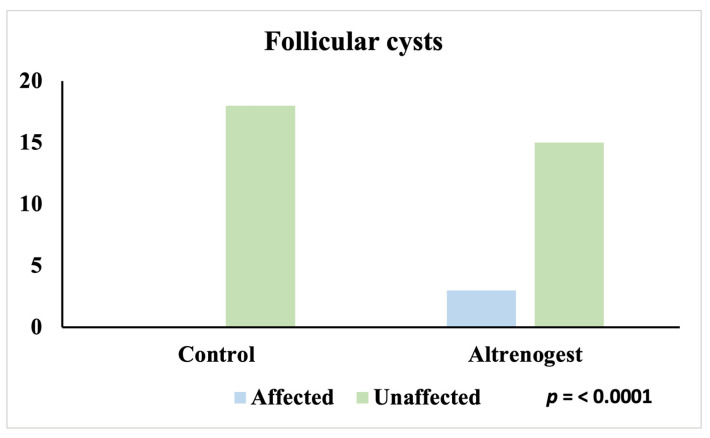
Incidence of follicular cysts in Control and Altrenogest groups.

A similar pattern emerged for follicular cysts, which were absent in the Control group and present only in the Altrenogest group (3/18; 17%) (*p* < 0.0001) (Figure 5).

Table 3 shows the age of the dolphins affected by pyometra and follicular cysts and the duration of treatment with Altrenogest before the onset of the diseases.

Regarding behavioral data, trainers reported no changes in play activities in conditioned animals, except for those during the period in which pyometra was diagnosed. Follicular cysts did not induce any behavioral changes.

## 4. Discussion

The aim of this study was to evaluate the effects of prolonged use of Altrenogest as a contraceptive method in bottlenose dolphins, using data obtained from ultrasound, hormonal, and behavioral monitoring. It is known that Altrenogest is a synthetic progestin used for its ability to inhibit the hypothalamic–pituitary–gonadal (HPG) axis through negative feedback, resulting in a reduction in GnRH secretion [13]. Its main use is related to the synchronization of estrus in gilts and primiparous sows [15,16], and it is also used in mares both to synchronize estrus and to predict its onset [16,17,18]. The use of Altrenogest has also been documented in cetaceans for the long-term suppression of ovulation [5] and to synchronize estrus in killer whales (*Orcine orca*), Pacific white-sided dolphins (*Lagenorhynchus obliquidens*), and bottlenose dolphins [13]. The data obtained in this study confirm the treatment’s efficacy in suppressing cyclic activity in bottlenose dolphins. Indeed, none of the treated females displayed overt sexual behavior, nor did any mating occur during the treatment period, indicating that the suppressive effect on the behavioral manifestation of estrus was effective. Regarding hormonal monitoring, treated subjects had statistically significantly lower serum progesterone levels than untreated subjects. This suggests effective suppression of the pre-ovulatory LH surge, responsible for ovulation [17], in subjects treated with Altrenogest. With ovulation inhibition, the corpus luteum does not form, and consequently, progesterone concentrations remain at basal levels. In contrast, in untreated subjects, concentrations show considerable variability and are higher, correlating with the fluctuations in normal cycles. Estrogen levels did not show statistically significant differences between treated and untreated animals, suggesting that estrogen production, regulated primarily by FSH through follicular growth, was not substantially inhibited. The presence of physiological estrogen levels in the absence of subsequent ovulation and progesterone production may represent an endocrine imbalance, thereby predisposing the subjects to side effects. The absence of CL and, therefore, the secretion of progesterone, a key hormone in protecting the endometrium and regulating the estrous cycle, can lead to prolonged uterine exposure to estrogens, a condition known to promote the onset of ovarian cysts, endometrial hyperplasia, and pyometra [19]. Studies conducted in pigs and horses have, in fact, shown that Altrenogest can cause behavioral alterations and long-term fertility problems [20]. One study showed that in prepubertal and mature gilts treated with Altrenogest, it increased the number of large antral follicles and, hence, predisposition to follicular cysts [20]. Another study demonstrated a drug-induced alteration in the immune system in non-pregnant mares. It is known that progesterone receptors are present in a variety of tissues and cell types, including lymphocytes. It was, therefore, hypothesized that progestin increased the expression and production of numerous pro-inflammatory cytokines, the increase of which would activate the NF-κB signaling pathway and increase the pathogen load in the reproductive tract [16]. In the present study, four dolphins treated with Altrenogest were found to have pyometra, while three others were diagnosed with follicular cysts. These findings may be related to the hormonal conditions highlighted. The etiopathogenesis of pyometra, in fact, is closely linked to the persistence of estrogenic stimuli on the endometrium in the absence of progestin balance. Estrogens promote the proliferation of endometrial glands and increase receptivity to inflammatory mediators, making the uterine environment more susceptible to ascending bacterial infections. Subsequent bacterial colonization and an inadequate local immune response can lead to the accumulation of purulent exudate within the uterus, resulting in a clinical picture of pyometra [19,21]. Furthermore, as mentioned, in the present study, ultrasound examination revealed the presence of follicular cysts in three dolphins. The etiopathogenesis of this condition can be traced back to the administration of synthetic progestin, which selectively inhibits the hypothalamic–pituitary–gonadal axis (especially LH), preventing ovulation while not interfering with FSH levels. Consequently, the follicles continue to develop under estrogen stimulation but never reach ovulation, resulting in follicular persistence and cyst formation [19]. It is important to note that one of the three females in which follicular cysts were observed and one of the females with pyometra had not undergone prolonged treatment with Altrenogest, but they were among the oldest females included in the study (57 years and 51 years, respectively). Therefore, advanced age could represent a predisposing factor.

## 5. Conclusions

The results obtained show that Altrenogest is an effective contraceptive treatment in dolphins. However, the data collected highlight that prolonged use of Altrenogest may predispose dolphins to the development of reproductive system disorders, particularly pyometra and follicular cysts, conditions observed in some of the treated females. These results suggest that, while it represents a potentially useful strategy for reproductive control in complex management contexts, its use requires careful balance between the desired effects and adverse effects.

## Figures and Tables

**Table 1:** Effect of group on estradiol and progesterone levels.

Parameter	Group		SEM	*p*-Value
	Control	Altrenogest		
Estradiol	9.03 ^A^	14.11 ^B^	0.63	<0.0001
Progesterone	3.01 ^A^	0.31 ^B^	0.29	<0.0001

SEM, standard error of the means. Different letters within the same row indicate significant differences between groups: ^A,B^ = *p* < 0.001.

**Table 2:** Effect of group, sampling time, and their binary interaction on estradiol and progesterone levels.

Group	Time											SEM	*p*-Value		
	0	6m	1y	1y6m	2y	2y6m	3y	3y6m	4y	4y6m	5y		G	T	G × T
	**Estradiol**														
Control	9.86 ^X^ (*n* = 18)	9.54 ^X^	9.38 ^X^	9.03 ^X^	8.61 ^X^	8.69 ^X^	9.57 ^X^	8.97 ^X^	8.74 ^X^	8.54 ^X^	8.46 ^X^	2.09	<0.0001	0.9935	0.9993
Altrenogest	14.91 ^Y^ (*n* = 18)	13.55 ^Y^	13.82 ^Y^	13.00 ^Y^	13.11 ^Y^	13.17 ^Y^	15.28 ^Y^	16.35 ^Y^	15.47 ^Y^ (*n* = 17)	13.23 ^Y^ (*n* = 17)	13.29 ^Y^ (*n* = 14)				
	**Progesterone**														
Control	1.50 ^X^ (*n* = 18)	3.57 ^X^	4.21 ^X^	3.79 ^X^	7.53 ^X^	3.05 ^X^	1.62 ^X^	1.18 ^X^	2.02 ^X^	3.01 ^X^	1.69 ^X^	0.97	<0.0001	0.0782	0.0731
Altrenogest	0.29 ^Y^ (*n* = 18)	0.31 ^Y^	0.34 ^Y^	0.29 ^Y^	0.29 ^Y^	0.24 ^Y^	0.26 ^Y^	0.29 ^Y^	0.33 ^Y^ (*n* = 17)	0.34 ^Y^ (*n* = 17)	0.42 ^Y^ (*n* = 14)				

SEM, standard error of the means; G, Group; T, Time. Different letters on the same column show statistical differences between groups at the same time: ^X,Y^ = *p* < 0.0001.

**Table 3:** Age and the duration of treatment with Altrenogest prior to diagnosis of pathology of affected dolphins.

ID_Dolphin	Age (Years)	Pathologies	Duration of Treatment with Altrenogest Prior to Diagnosis of Pathology (Years)
A1	42	Pyometra	7
A2	57	Follicular Cyst	2
B1	29	Follicular Cyst	5
B3	52	Pyometra	5
B6	17	Follicular Cyst	9
C3	50	Pyometra	2
D1	16	Pyometra	7

## Data Availability

The raw data supporting the conclusions of this article will be made available by the authors on request.
